# Metastatic neuroendocrine carcinoma of right adrenal gland successfully treated with laparoscopic adrenalectomy after multimodal therapy

**DOI:** 10.1002/iju5.12511

**Published:** 2022-07-29

**Authors:** Yusuke Yamagata, Takashige Abe, Naoya Iwahara, Kohichi Takada, Yasuhiro Hida, Emi Takakuwa, Hiroshi Kikuchi, Ryuji Matsumoto, Takahiro Osawa, Nobuo Shinohara

**Affiliations:** ^1^ Department of Urology Hokkaido University Graduate School of Medicine Sapporo Japan; ^2^ Department of Medical Oncology Sapporo Medical University School of Medicine Sapporo Japan; ^3^ Department of Cardiovascular and Thoracic Surgery, Faculty of Medicine Hokkaido University Sapporo Japan; ^4^ Department of Surgical Pathology Hokkaido University Hospital Sapporo Japan

**Keywords:** adrenal gland neoplasms, adrenalectomy, combined modality therapy, laparoscopic surgery, metastasectomy, salvage therapy

## Abstract

**Introduction:**

We report a case of laparoscopic adrenalectomy in a salvage setting after multiple chemotherapies for neuroendocrine carcinoma.

**Case presentation:**

A 49‐year‐old man was diagnosed with unknown primary carcinoma with single brain metastasis, and right supraclavicular and mediastinal lymph node metastases. After stereotactic radiotherapy of the brain metastasis and systemic chemotherapy, lymphadanectomy was performed. The pathologic diagnosis was neuroendocrine carcinoma. At 11 months after surgery, computed tomography revealed right adrenal metastasis. Local radiotherapy initially resulted in complete remission. However, adrenal recurrence was noted 10 months later. Laparoscopic adrenalectomy was performed with curative intent. The patient is currently alive without recurrence at 20 months after the operation.

**Conclusion:**

Adrenalectomy can become a treatment option if other metastases are well‐controlled with systemic therapy. Surgical elimination of oligometastases can offer long‐term disease control in selected patients as part of a multimodal approach.

**Keynote message**

We report a case of laparoscopic adrenalectomy for adrenal recurrence of neuroendocrine carcinoma after multiple chemotherapies and local radiotherapy. Surgical disease elimination can become a treatment option if other metastatic sites are well‐controlled with systemic therapy.

## Introduction

In patients with a history of malignancy, metastases to the adrenal gland is a possibility. In general, synchronous occurrence is a dominant form of disease presentation, and systemic therapy is a mainstay treatment for disseminated disease. Although isolated adrenal metastasis remains uncommon, previous studies revealed that surgical elimination contributed to long‐term disease control in selected patients.^1^, ^2^, ^3^, ^4^, ^5^ Herein, we report a case of adrenal recurrence of a neuroendocrine tumor after systemic chemotherapy and local radiotherapy, that was successfully treated by laparoscopic adrenalectomy.

## Case presentation

**Fig. 1:**
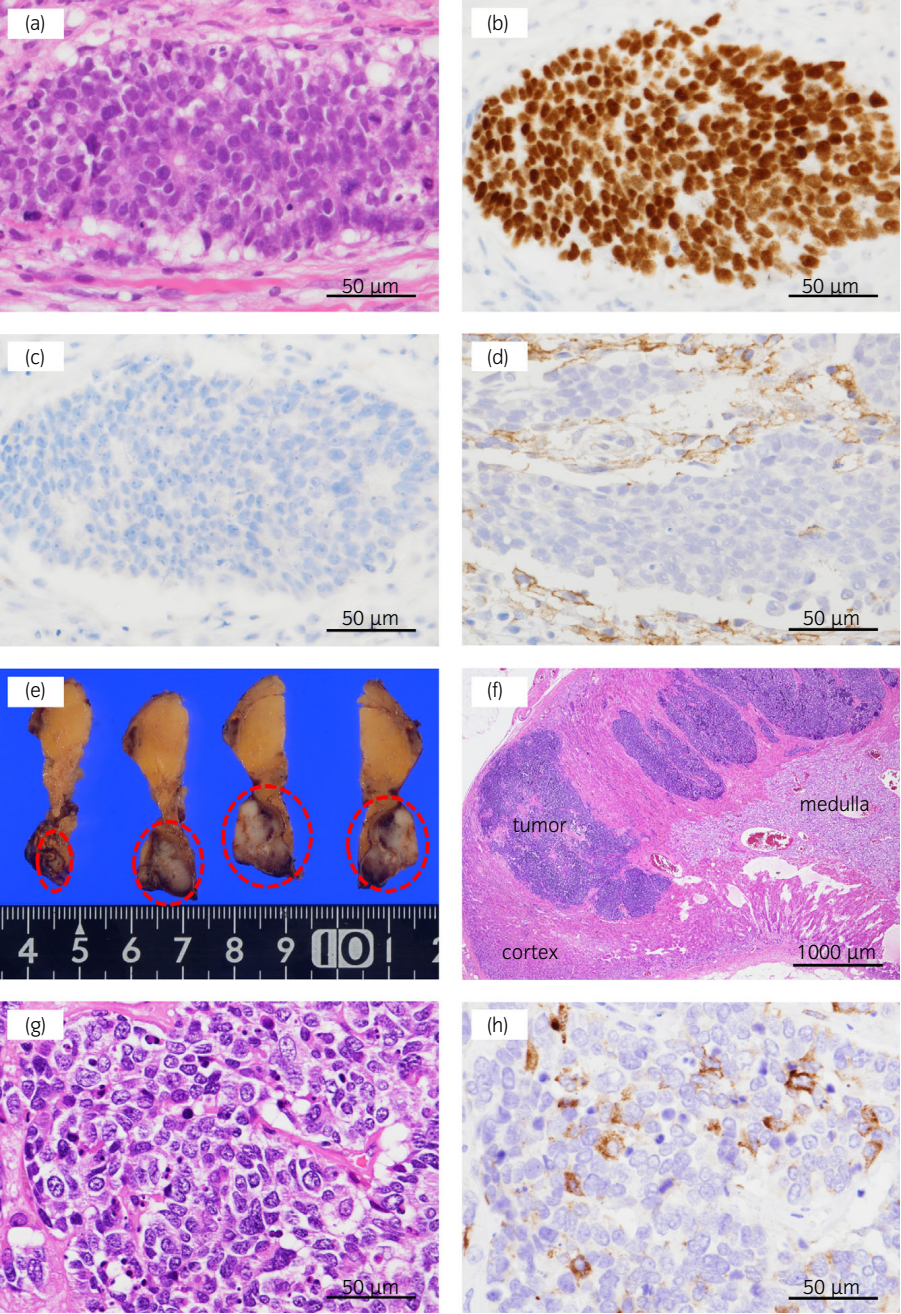
(a)‐(d) Pathologic findings of the right supraclavicular lymph node biopsy specimen (a) Hematoxylin and eosin staining (b) TTF‐1 is positive. (c) ALK is negative. (d) PD‐L1 is weak positive. (e)‐(h) Pathologic findings of the right adrenal tumor (e) A well‐circumscribed white lesion is found in the right adrenal gland. (red circle). (f) Low‐magnification view of the right adrenal gland. (g) Hematoxylin and eosin staining (h) Chromogranin A, which is specific for neuroendocrine tumors, is partially positive.

A 49‐year‐old male presented to the previous hospital with the complaint of an anterior neck mass. He did not have a significant medical history, other than being a current smoker. FDG Positron emission tomography (PET)‐computed tomography (CT) revealed right supraclavicular and mediastinal lymph node swellings. As single brain metastasis was also detected in the right parietal lobe on magnetic resonance imaging (MRI), although the primary site remained unknown regardless of additional lung and abdominal CT imaging using radiocontrast. Regarding serum tumor markers, ProGRP was elevated to 676 pg/mL (normal range: 0–80.9 pg/mL), while NSE was within the normal range. Supraclavicular lymph node biopsy revealed that cancer cells were TTF‐1‐positive, ALK‐negative, expressed a low PD‐L1 on immunohistochemistry, and were EGFR mutation‐negative on molecular analysis (Fig. 1a‐d). As an unknown primary carcinoma with the most likely diagnosis being non‐small cell lung carcinoma, a combined regimen of cisplatin, pembrolizumab, and bevacizumab was initiated. Stereotactic radiotherapy was also administered for the brain metastasis (32 Gy/4 fractions). After three courses of the systemic therapy, the lymph node metastases showed partial response (PR), and the brain metastasis was well‐controlled without new disease development. Subsequently, right supraclavicular and mediastinal lymph node dissection was carried out by thoracic surgeons at our hospital. The pathology was neuroendocrine carcinoma, based on histologic characteristics and immunohistochemical positives of TTF‐1, synaptophysin, chromogranin A, and CD56. Adjuvant chemotherapy (cisplatin and etoposide, 1 cycle) and radiotherapy to the surgical area (right supraclavicular area, 56 Gy; mediastinum area, 60 Gy) were added postoperatively.

**Fig. 2:**
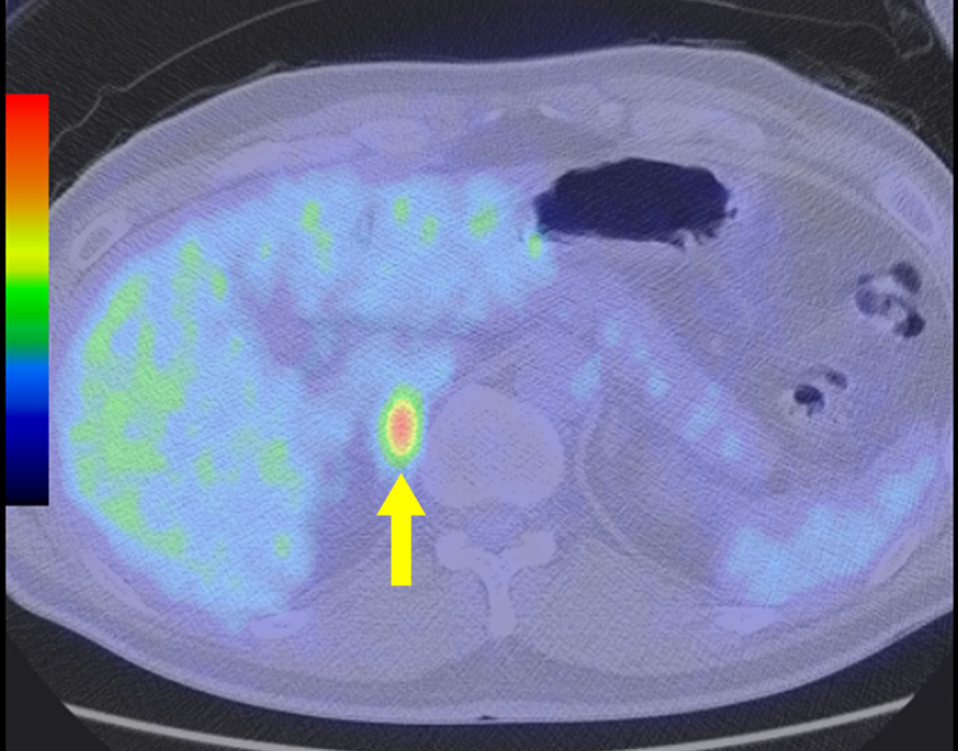
FDG PET‐CT findings. Fluorodeoxyglucose uptake is shown in the right adrenal gland at 11 months after right supraclavicular and mediastinal lymph node dissection.

**Fig. 3:**
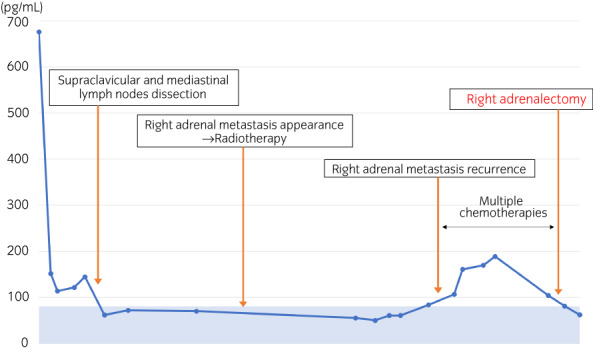
Transition of ProGRP during the treatment (normal range: 0–80.9 pg/mL).

**Fig. 4:**
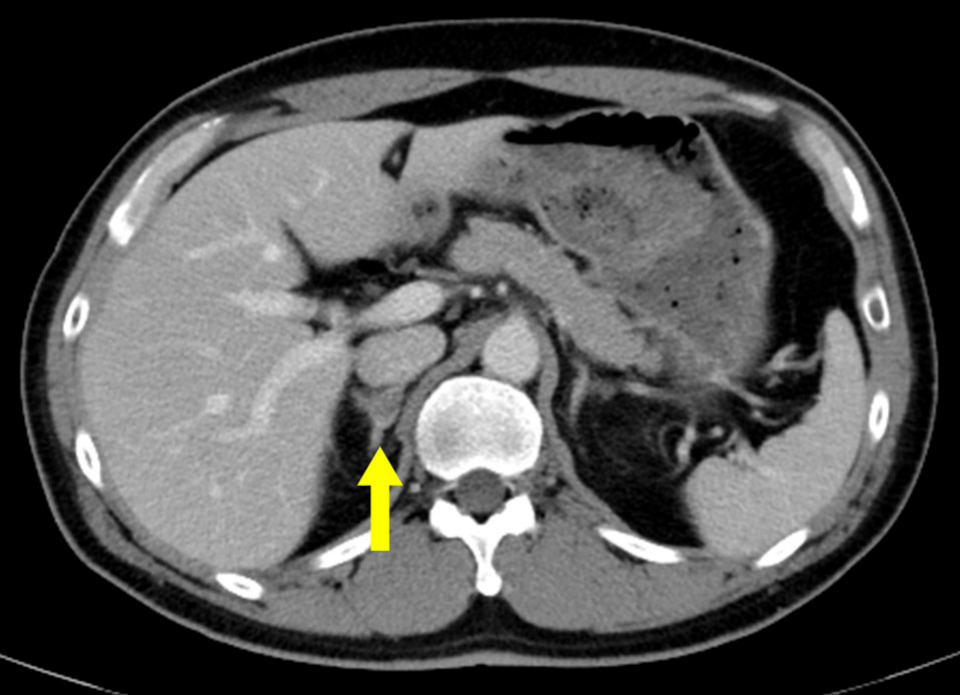
Enhanced computed tomography before adrenalectomy. Right adrenal metastasis remains after local radiotherapy and multiple chemotherapies.

At 11 months after surgery, PET‐CT revealed right adrenal metastasis (Fig. 2), and local radiotherapy (48 Gy) resulted in complete remission (CR). However, right adrenal metastasis recurred at 10 months after radiotherapy, and pembrolizumab was re‐administered. During the treatment, the serum ProGRP level gradually increased from 92.9 to 104.3 pg/mL (Fig. 3), and the right adrenal gland also enlarged. Amrubicin was utilized as salvage therapy, and a PR was observed after two courses of treatment. The patient was referred to our department, aiming for surgical CR. Figure 4 shows a 16 × 11‐mm right adrenal mass on preoperative CT.

Under general anesthesia, the patient was placed in the left lateral position. Laparoscopic adrenalectomy (transperitoneal approach) was performed via four ports. Severe adhesion was noted around the adrenal central vein. This area could be dissected by sharp dissection (Video S1). The adrenal gland was completely resected macroscopically. The operative time was 151 minutes, and blood loss was minimal. No perioperative complications occurred, and the postoperative recovery was uneventful. The patient was discharged 4 days after surgery. Pathologic examination revealed the tumor was metastatic neuroendocrine carcinoma, with a positive margin in a small area (Fig. 1e‐h). After surgery, 2 cycles of amrubicin were added in an adjuvant setting. Now, at 20 months after surgery, the patient is alive without recurrence.

## Discussion

In the present case, although the primary site was unknown, systemic combination therapy, radiotherapy for brain metastasis, and subsequent surgery for lymph node metastases were successful as initial treatments. Adrenal metastasis occurred at 11 months after surgery, although the aforementioned other sites were well‐controlled. Because he did not develop new lesions other than adrenal metastasis during the subsequent treatment, we performed laparoscopic adrenalectomy, which resulted in the long‐term disease control that has been achieved so far. We believe that disease stability during systemic treatment should be one of the key factors when physicians consider metastasectomy for oligometastases. The guideline published by the American Association of Clinical Endocrinologists and American Association of Endocrine Surgeons also states that surgical resection can improve disease‐free survival in selected patients with good control of extra‐adrenal disease and a good performance status.^6^

So far, favorable outcomes of laparoscopic adrenalectomy for isolated adrenal metastasis have been accumulated.^1^, ^2^, ^3^, ^4^, ^5^ Goto et al. reported in their retrospective multicenter study that in 67 patients who underwent laparoscopic adrenalectomy for metastatic adrenal mass, lung cancer (30%) and renal cell carcinoma (30%) were the most frequent primary sites, 72% (48/67) presented with isolated adrenal metastasis, and the median overall survival was 3.8 years.^3^ Based on preoperative CT, which indicated a surgical layer between the tumor and vena cava, we also selected a laparoscopic approach. To our knowledge, this is the first reported case of laparoscopic adrenalectomy in a salvage setting after local radiotherapy. As aforementioned, fibrous reactive tissue was observed around the central vein, and meticulous sharp dissection was required. In urologic cancer surgery, salvage radical prostatectomy for recurrence after radiation therapy has been performed mainly in Western high‐volume centers. Historically, open salvage radical prostatectomy was associated with a high risk of complications, poor functional outcomes, and dismal cancer control.^7^, ^8^ Recently, robotic surgery has been rapidly adopted in this setting, leading to significant improvements in perioperative, functional, and oncologic outcomes.^9^, ^10^ Possible advantages associated with a robotic approach are decreased blood loss due to pneumoperitoneum, magnification of the surgical view by high‐resolution cameras, and meticulous manipulation by multi‐articulated robotic arms. Regarding the aids of pneumoperitoneum and high‐resolution images, they should also be helpful in conducting salvage laparoscopic adrenalectomy after local radiotherapy. At present, in Japan, robotic adrenalectomy has not been approved by the national health care system.

Regarding the surgical elimination in metastatic neuroendocrine tumor, Ochiai T et al. reported a case of right adrenal neuroendocrine tumor with multiple celiac lymph node metastases and solitary liver metastasis.^11^ After the 14 courses of systemic chemotherapy (cisplatin plus irinotecan), all the lesions became more than 50% tumor response. Surgical elimination (adrenalectomy, hepatectomy, and lymphadenectomy) was performed, and the patient was disease‐free for 9 months after surgery. As abovementioned, we underscore that good response of systemic chemotherapy and disease stability are crucial when considering metastasectomy.

There are several limitations of the current study. The follow‐up time remains short. Because we did not find other reports of salvage adrenalectomy after local radiotherapy, its indication should be thoroughly discussed among multidisciplinary physicians, and surgery should be performed by an expert laparoscopic surgeon with sufficient experience.

## Conclusion

Adrenalectomy may become a treatment option if other metastatic sites are well‐controlled with systemic therapy. Surgical elimination of oligometastases can offer long‐term disease control in selected patients as part of a multimodal approach.

## Author contributions

Yusuke Yamagata: Visualization; writing – original draft. Takashige Abe: Project administration; supervision; writing – review and editing. Naoya Iwahara: Supervision; writing – review and editing. Kohichi Takada: Writing – review and editing. Yasuhiro Hida: Writing – review and editing. Emi Takakuwa: Resources; writing – review and editing. Hiroshi Kikuchi: Writing – review and editing. Ryuji Matsumoto: Writing – review and editing. Takahiro Osawa: Writing – review and editing. Nobuo Shinohara: Writing – review and editing.

## Conflict of interest

The authors declare no conflict of interest.

## Approval of the research protocol by an institutional reviewer board

N/A.

## Informed consent

Informed consent was obtained from the patient.

## Registry and the registration no. of the study/trial

N/A.

## Supporting information


**Video S1** Edited surgical video. Two 12‐mm trocars for the surgeon and one 5‐mm trocar for the assistant were placed on the right side along the costal arch (0:00). Fibrous tissue surrounded the central vein and vena cava (0:10). The central vein was clipped twice and cut on the peripheral side (0:47). The tumor was separated from the kidney along the upper pole (1:14). The head side of the tumor also adhered firmly and required sharp detachment (1:20). The tumor was completely excised (1:44).
